# Novel oxidative stress-related prognostic biomarkers for melanoma associated with tumor metastasis

**DOI:** 10.1097/MD.0000000000024866

**Published:** 2021-02-26

**Authors:** Xianpei Wu, Jinmin Zhao

**Affiliations:** a Department of Orthopedics Trauma and Hand Surgery; b Guangxi Engineering Center in Biomedical Materials for Tissue and Organ Regeneration; c Guangxi Collaborative Innovation Center for Biomedicine; d Guangxi Key Laboratory of Regenerative Medicine, The First Affiliated Hospital of Guangxi Medical University, Nanning, Guangxi Zhuang Autonomous Region, P.R. China.

**Keywords:** integrated bioinformatics analysis, metastatic, oxidative stress, prognosis, skin cutaneous melanoma

## Abstract

Skin cutaneous melanoma (SKCM) is a prevalent skin cancer whose metastatic form is dangerous due to its high morbidity and mortality. Previous studies have systematically established the vital role of oxidative stress (OS) in melanoma progression. This study aimed to identify prognostic OS genes closely associated with SKCM and illustrate their potential mechanisms. Transcriptome data and corresponding clinical traits of patients with SKCM were retrieved from The Cancer Genome Atlas and Gene Expression Omnibus databases. A weighted gene co-expression network analysis was conducted to identify relationships between clinical features and OS genes in specific modules. Subsequently, Cox regression analysis was performed on candidate OS genes; four hub prognosis-associated OS genes (AKAP9, VPS13C, ACSL4, and HMOX2) were identified to construct a prognostic model. After a series of bioinformatics analysis, our prognostic model was identified significantly associated with the overall survival of patients with SKCM and metastatic ability of the cancer. Furthermore, our risk model demonstrated improved diagnostic accuracy in the Cancer Genome Atlas and Gene Expression Omnibus cohorts. In addition, we established 2 nomograms based on either risk score or hub genes, which displayed favorable discriminating ability for SKCM. Our results provide novel insight into the potential applications of OS-associated genes in SKCM.

## 1 Introduction

Skin cutaneous melanoma (SKCM), a highly aggressive malignant tumor, is a serious threat to human health.^[1]^ According to the GLOBOCAN database, 287,723 new patients with melanoma were diagnosed worldwide in 2018, of which 21.1% died.^[2]^ Occurrence and progression of SKCM are significantly associated with the degree of skin pigmentation. Indeed, patients with low skin pigmentation content exposed to ultraviolet radiation comprise the majority of SKCM cases.^[3,4]^ Metastatic SKCM, with high rates of metastasis,^[5]^ is the most common form and is regarded as the leading cause of skin cancer deaths.^[6]^ Globally, the 10-year overall survival of patients with stage I and II SKCM ranges from 98% to 75%,^[7]^ but one third of patients will progress to metastatic melanoma,^[8]^ making early diagnosis and individualized treatments vital. Currently, the optimal management of metastatic melanoma comprises targeted therapy and checkpoint suppression.^[9,10]^ However, therapeutic effectiveness remains limited due to lack of knowledge regarding the intricate mechanism underlying SKCM metastasis. In recent years, many studies have focused on identifying potential biomarkers for metastatic melanoma.^[11,12]^ Unfortunately, these developments have been inadequate for improving early diagnosis and prognosis of SKCM. Thus, development of more effective biomarkers is urgently needed for accurate identification and prognostication of SKCM.

The unequivocal mechanism leading to SKCM metastasis remains poorly understood; however, oxidative stress (OS) has been proposed as an important factor driving tumorigenesis and cancer progression through excessive production of reactive oxygen species (ROS).^[13–15]^ Imbalances between antioxidants and oxidants favor the oxidants, which potentially leads to biological damage, termed OS. As a characteristic of OS, ROS comprise a number of free radicals or reactive nonradical species, including singlet oxygen, hydrogen peroxide, superoxide anion, and so on,^[16]^ which are dramatically elevated in patients with SKCM.^[17]^ Excessive ROS presence leads to genotoxicity and DNA damage when scavenging potential is outrun,^[18,19]^ eventually inducing various genomic mutations and initiating tumorigenesis.^[20,21]^ In melanocytes, ROS are mainly derived from melanosomes, NADPH oxidase family of enzymes, mitochondria, nitric oxide synthase activity, and several arachidonic acid oxygenase activities.^[22]^ Interestingly, the relationship between melanin and ROS appears to be a double-edged sword. On the 1 hand, melanin can protect melanocytes from UV radiation, but its synthesis in epidermal melanocytes results in additional intracellular ROS generation, which is also 1 of the reasons that melanocytes are particularly susceptible to OS.^[23,24]^ Together, these studies indicate that OS is closely associated with the progression of SKCM. Nevertheless, the value of OS genes in SKCM prognosis prediction remains largely unclarified and its underlying mechanisms require further validation.

High-throughput technologies have been widely applied to illustrate connections between hub genes and SKCM.^[25,26]^ A weighted gene co-expression network (WGCN) is frequently constructed to describe gene association patterns between different phenotypic traits.^[27]^ SKCM data can thus be used to construct a powerful scale-free network to identify hub biomarkers or therapeutic targets for prognosis evaluation and clinical diagnosis,^[28,29]^ with virtual applications in pathological or biological process identification (such as tumor progression), as well as brain imaging data and genetics analyses.^[30]^ In the present study, we identified key modules and hub OS genes associated with the prognosis of and metastasis in patients with SKCM using WGCN analysis (WGCNA) to construct a risk model. We also applied a series of systematic analyses to identify the function and clinical significance of each identified gene in patients with SKCM, which may provide potential prognostic and diagnostic biomarkers for SKCM.

## 2 Materials and methods

### 2.1 Data acquisition

An RNA-sequencing dataset containing 471 SKCM samples and 1 normal skin tissue sample was downloaded from The Cancer Genome Atlas (TCGA) (https://portal.gdc.cancer.gov); meanwhile, its corresponding clinical and prognostic parameters were acquired from the University of California Santa Cruz Xena (http://xena.ucsc.edu/).^[31]^ Transcriptome data of 812 whole skin samples were retrieved from the Genome Tissue Expression database (https://gtexportal.org/home/datasets).^[32,33]^ Additionally, gene expression profiles and clinical information of 214 patients with SKCM from the Gene Expression Omnibus GSE65904 corhort (https://www.ncbi.nlm.nih.gov/geo/) were downloaded as a validation cohort.^[34]^ All gene expression files were log2-transformed, and profiles from different platforms were normalized using the R package “sva” to remove batch effects reported by previous studies.^[35,36]^ Furthermore, 1399 protein domains of OS were downloaded from the GeneCards database (https://www.genecards.org) with relevance score ≥ 7, and subsequently applied for further exploration.

### 2.2 Construction of WGCN and hub gene identification

In this study, a WGCN was constructed to explore the relationship between expression modules and numerous clinicopathological features (overall survival time, overall survival state, age, gender, tumor stage, and metastasis). Expression profiles of OS genes in TCGA cohort were utilized to create a co-expression network with R package “WGCNA”,^[37,38]^ and pairwise Pearson's correlation coefficients were calculated between genes. Two parameters [module eigengenes and gene significance (GS)] were identified to reveal modules most relevant to SKCM overall survival and metastasis. Hub genes comprising highly interconnected nodes within the module were regarded as functionally significant.^[39]^ Thus, after choosing a significant module, genes with high module membership (MM) (MM > 0.6 and GS > 0.2) was selected. Transcriptional expression levels were then compared in normal tissues and SKCM samples; significantly differentially expressed genes were defined as candidate OS genes.

### 2.3 Gene ontology (GO) and kyoto encyclopedia of genes and genomes (KEGG) enrichment analyses

GO enrichment and KEGG pathway analyses were applied to systematically investigate the biological functions of OS genes in key modules using Database for Annotation, Visualization, and Integrated Discovery version 6.8.^[40]^ GO analysis comprehensively comprised 3 terms: biological process, cellular component, and molecular function. Both *P* and false discovery rate values <.05 were considered statistically significant.

### 2.4 Prognostic model construction and efficacy evaluation

All candidate OS genes were subjected to univariate Cox regression analysis with R package “survival” to explore the relationship between each gene and patient overall survival; genes with *P* < .05 were identified as prognosis-related OS genes. Thereafter, these genes were integrated to construct a multivariate Cox proportional hazards regression model, categorizing patients with SKCM into high or low risk groups. The formula of each sample's risk score was calculated as follows:

risk score=Σexpgenei*βi

where expgenei represents the relative expression value of OS gene i, and β represents the regression coefficient.

The Kaplan–Meier method and log-rank test using R/Bioconductor package “survival” were further conducted to compare outcomes between 2 risk subgroups. Additionally, the R package “survivalROC” was used to validate the predictive accuracy of the gene signature.^[41]^ Univariate and multivariate Cox regression analyses were also conducted to evaluate the relationship between clinical characteristics and risk score. Finally, a nomogram incorporated with calibration plots was constructed to forecast the clinical outcome of patients with SKCM using R package “rms”.^[42]^

### 2.5 Hub genes evaluation

To clarify the differential expression of the hub OS genes at a protein level, data in the Human Protein Atlas (HPA) online database (http://www.proteinatlas.org/) was used to elucidate the differences between normal and SKCM tumor tissues.^[43]^ Expression of these OS genes in SKCM was also verified in TCGA dataset. Furthermore, to calculate the prognostic value of each gene for SKCM, the Kaplan-Meier method was employed on TCGA cohort, and the relationship between hub gene expression and different clinicopathological features of SKCM was systematically compared, especially connections with the cancer's metastatic abilities. Nomogram and calibration plots based on the expression of hub genes were also constructed to forecast the clinical outcome of patients with SKCM.

### 2.6 Gene set enrichment analysis (GSEA)

To explore the potential functional roles that were significantly enriched in our constructed prognostic gene signature, GSEA was applied to a curated gene set “c2.cp.kegg.v7.1.symbols.gmt” and a GO gene set “c5.all.v7.1.symbols.gmt.” A normalized *P* value < .05 and false discovery rate q value < 0.05 were considered statistically significant.

## 3 Results

### 3.1 Identification of hub module by constructing WGCN

**Figure 1:**
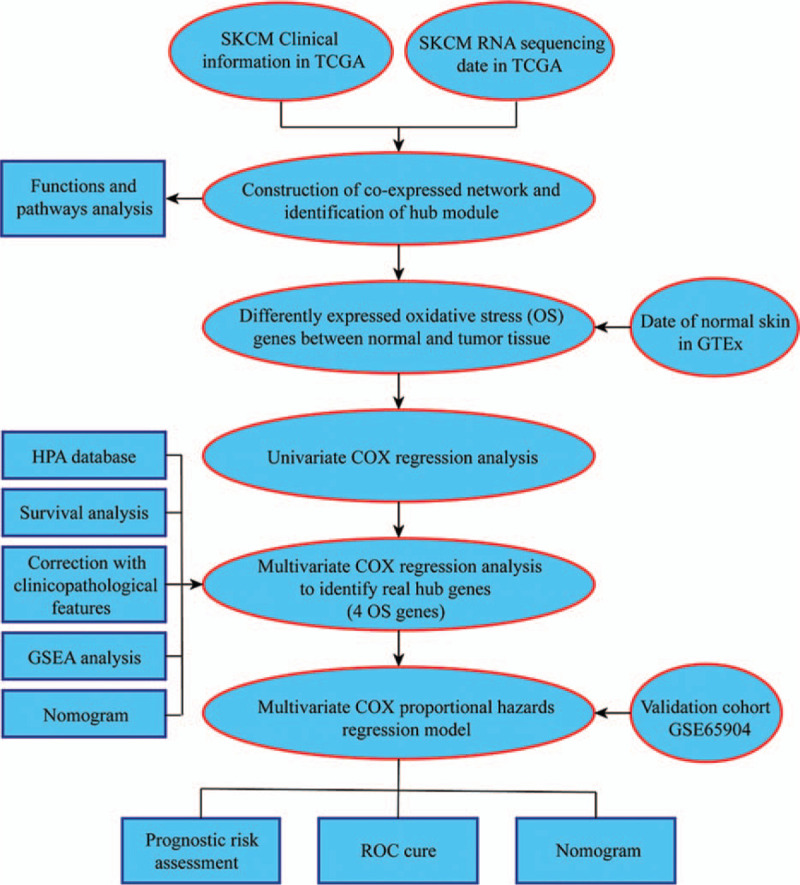
Flowchart describing the schematic overview of the study design.

**Figure 2:**
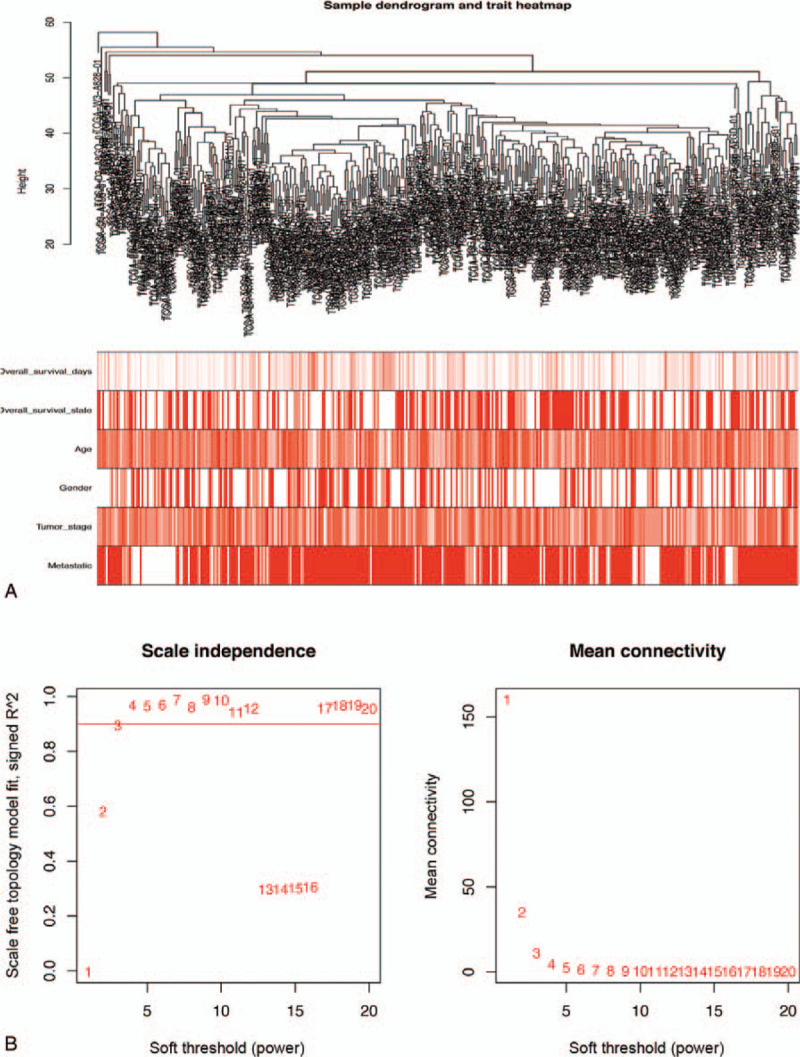
Determination of soft-thresholding power in the WGCNA. (A) Clustering dendrogram of 471 SKCM samples and associated clinical traits. (B) The scale-free fit index for soft-thresholding powers. SKCM = skin cutaneous melanoma, WGCNA = WGCN analysis.

**Figure 3:**
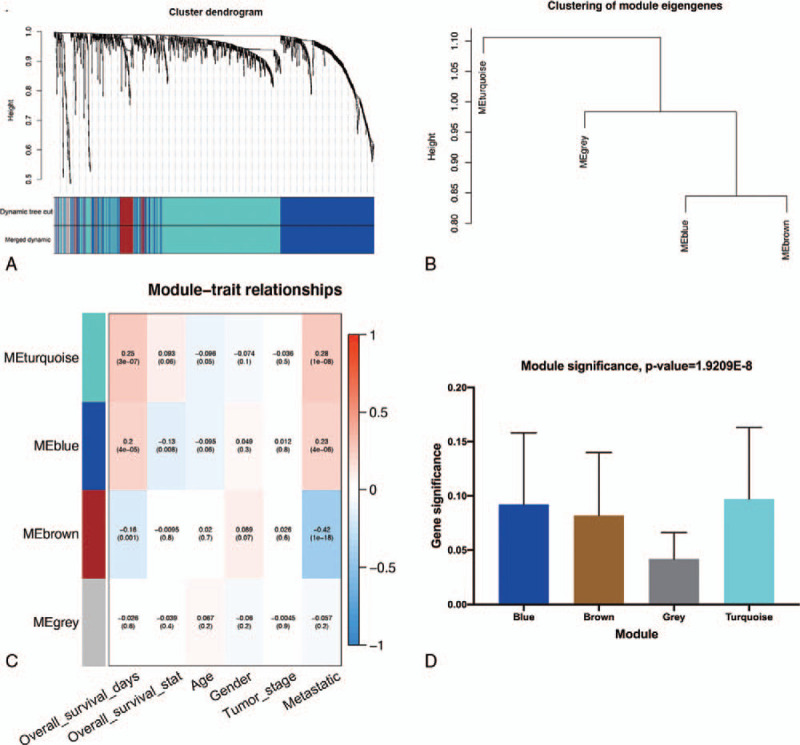
Identification of modules related to the overall survival and metastasis of SKCM. (A) A dendrogram of the differentially expressed genes clustered based on different metrics. (B) Clustering of modules eigengenes. (C) A heatmap showing the correlation between the gene module and clinical traits. The correlation coefficient in each cell represented the correlation between gene module and the clinical traits, which decreased in size from red to blue. (D) Distribution of average gene significance and errors in the modules associated with the overall survival of SKCM patients. SKCM = skin cutaneous melanoma.

Bioinformatics analysis of publicly available datasets was performed according to the flow chart shown in Figure 1. First, WGCNA of 471 SKCM samples with corresponding clinical data from TCGA cohort was carried out on 1399 extracted OS genes. As shown in Figure 2A, six clinical characteristics of patients with SKCM, including overall survival state, overall survival time, age, gender, tumor stage, and metastasis, were included for analysis. To construct a scale-free network, β = 3 (scale-free R2 = 0.89) was selected as the soft threshold (Fig. 2B), and a total of four co-expressed modules were identified (Fig. 3A–B). Subsequently, each module was assigned a different color to identify the module most associated with overall survival of SKCM. Among all modules, the turquoise module was specifically positively associated with tumor survival (*P* < .05). Interestingly, the 695 genes in the turquoise module also had the closest associations with SKCM metastasis (*P* < .05, Fig. 3C–D). Thus, the turquoise module was identified as the key module of interest in TCGA cohort.

### 3.2 Functional enrichment analysis

**Figure 4:**
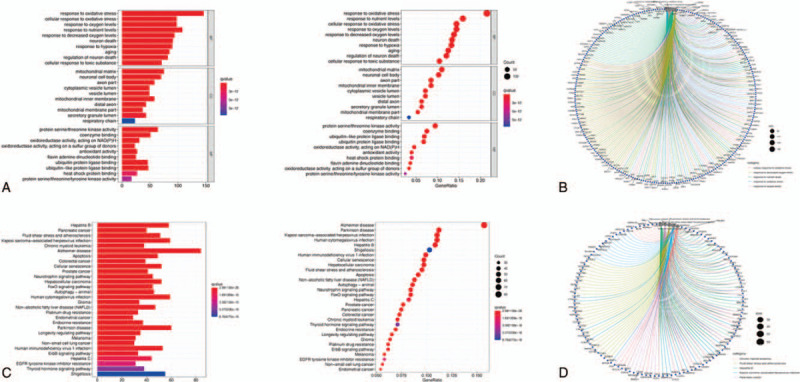
Functional enrichment analysis of turquoise module. (A) Top 10 classes of GO enrichment terms in biological process, cellular component (CC), and molecular function (MF). (C) Top 30 classes of KEGG enrichment terms. In each bubble plot, the size of the dot represents the number of enriched genes. Circle diagram of turquoise module genes which enriched in GO (B) and KEGG (D) analysis. KEGG = Kyoto Encyclopedia of Genes and Genomes.

GO enrichment analysis suggested that the 695 OS genes were mainly enriched in the biological process category, related to response to oxidative stress, oxygen levels, nutrient levels, hypoxia, and neuron death (Fig. 4A). In terms of molecular function, the key module was notably enriched in protein serine/threonine kinase activity, coenzyme binding, and oxidoreductase activity acting on NAD(P)H. With regard to the cellular component category, the key module was significantly enriched in mitochondrial matrix, neuronal cell body, and axon part. Gene symbols and their interaction with the enriched functions in GO are shown in Figure 4B. In addition, the results of KEGG enrichment analysis indicated that the 695 OS genes in the turquoise module were mainly enriched in pathways of Hepatitis B, pancreatic cancer, fluid shear stress and atherosclerosis, chronic myeloid leukemia, and Kaposi sarcoma-associated herpesvirus infection (Fig. 4C). OS genes enriched in these pathways are displayed in Figure 4D.

### 3.3 Screening of prognosis-related OS genes and construction of a genetic risk score model for patients with SKCM

**Figure 5:**
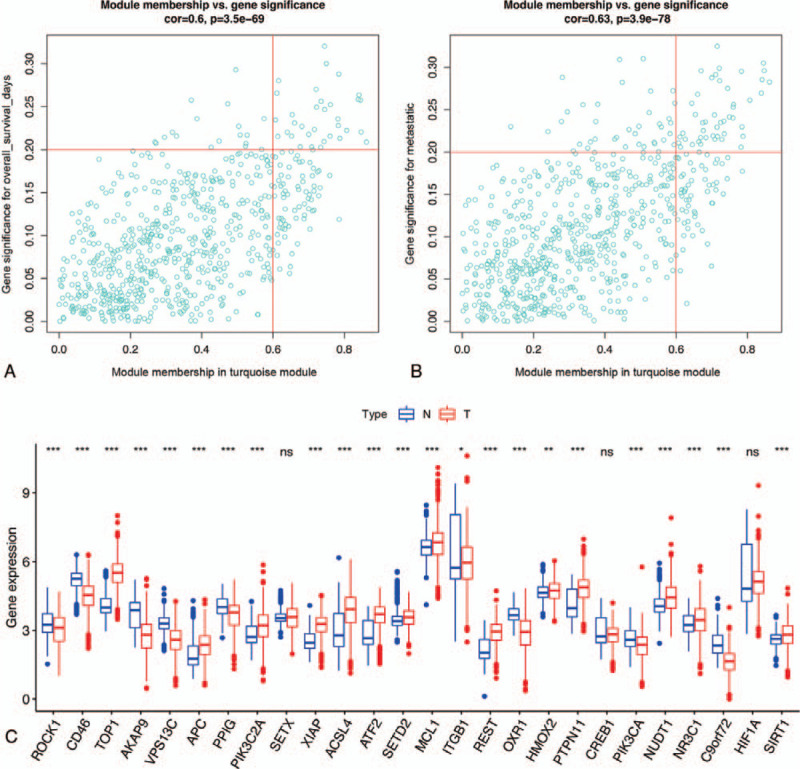
Identification of hub genes in turquoise module. Scatter plot of module eigengenes in exploring the connectivity with overall survival (A) and metastatic (B) of SKCM. (C) The mRNA expression pattern of selected OS genes in TCGA-SKCM cohort. SKCM = skin cutaneous melanoma, TCGA = the Cancer Genome Atlas.

**Figure 6:**
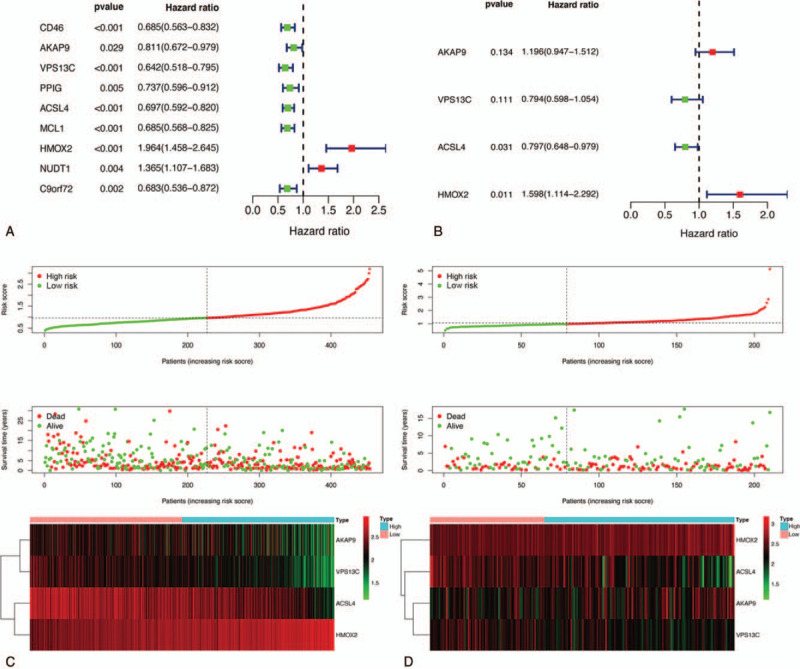
Construction of prognostic model in the TCGA and GSE65904 cohort. (A) Univariate Cox regression analysis for identification prognosis-associated OS genes. (B) Multivariate Cox proportional hazards regression model was constructed based on the identified prognostic-related OS genes. Risk score distribution, survival status, and expression heat map of TCGA (C) and GSE (D) cohort. TCGA = the Cancer Genome Atlas.

Twenty-six highly relevant OS genes were screened from the turquoise module (Fig. 5A–B). Subsequently, transcription expression levels of these genes were compared between SKCM samples and normal tissues (Fig. 5C). Ultimately, 23 significantly differentially expressed OS genes were identified as candidate SKCM metastasis- and prognosis-associated genes. These genes further underwent univariate Cox regression analysis to identify nine OS genes with *P* < .05 (Fig. 6A). Thereafter, a multivariate Cox proportional hazards regression model was constructed (Fig. 6B), and four OS genes (AKAP9, VPS13C, ACSL4, and HMOX2) were ultimately selected for calculation of the genetic risk score. Meanwhile, all patients with SKCM in TCGA or GSE65904 cohorts were separated into low- and high-risk groups according to the median risk score (Fig. 6C–D).

### 3.4 Evaluating prognostic value of hub OS genes in patients with SKCM

**Figure 7:**
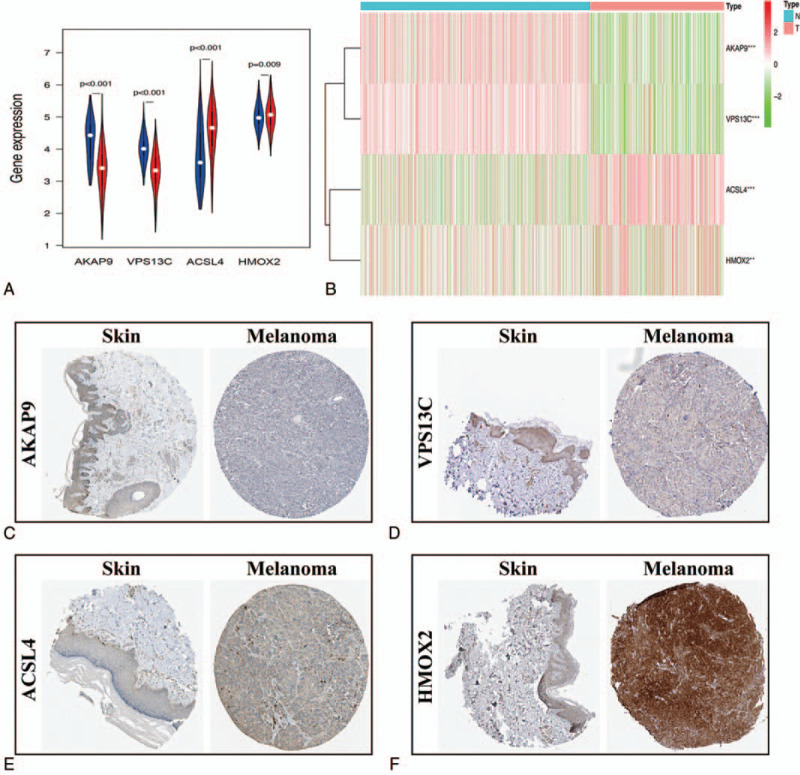
The expression of prognosis-associated OS genes in SKCM patients. The Violin plot (A) and heatmap (B) reveals the transcription expression of OS genes in TCGA cohort. HPA database verifies the protein expression of AKAP9 (C), VPS13C (D), ACSL4 (E), HMOX2 (F) in SKCM. SKCM = skin cutaneous melanoma.

**Figure 8:**
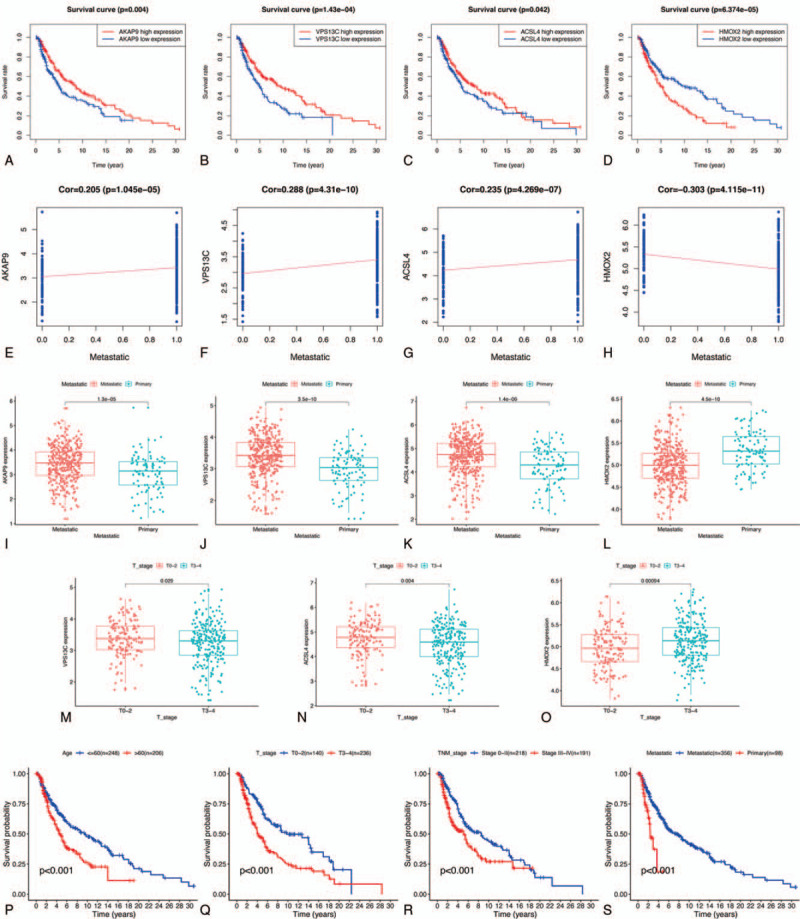
Evaluation the prognostic value of prognosis-related OS genes in TCGA-SKCM patients. (A-D) Survival curve of OS genes. (E-H) Correlation analysis between 4 progression-associated OS genes expression and cancer metastatic. (I-O) The relationship between 4 prognosis-associated OS genes expression and clinicopathological characteristics of SKCM. (P-S) Survival curve of clinicopathological characters. SKCM = skin cutaneous melanoma.

First, we extracted the expression value of each key gene from TCGA cohort and drew a violin plot and heatmap. As shown in Figure 7A–B, ACSL4 and HMOX2 were significantly overexpressed in SKCM samples, while expression of AKAP9 and VPS13C was decreased in comparison to that in normal tissues. Similar results were validated by analyzing the protein expression level of the key OS genes in accordance with the immunohistochemistry results from the HPA database (Fig. 7C–F). Subsequently, Kaplan-Meier analysis was also carried out to investigate the prognostic value of the four OS genes in SKCM. As shown in Figure 8A–D, increased overall survival was significantly associated with elevated expression of AKAP9 (*P* = .004), VPS13C (*P* = 1.43e-04), and ACSL4 (*P* = .042); however, HMOX2 (*P* = 6.374e-05) expression had the opposite effect on the prognosis of patients with SKCM. These results indicated that our four identified OS genes were significantly correlated with the prognosis of SKCM.

### 3.5 Validating the relationship between OS genes and clinicopathological features of SKCM

While investigating the association between expression of hub OS genes and SKCM clinical features, we discovered that patients with metastatic melanoma had significantly increased expression of AKAP9, VPS13C, and ACSL4 (Fig. 8I–K), while HMOX2 demonstrated higher expression in patients with primary melanoma (Fig. 8L). Therefore, we further investigated connections between AKAP9, VPS13C, ACSL4, and HMOX2 expression and cancer metastasis. Results indicated that the four hub genes were all significantly related to metastasis (*P* < .05; Fig. 8E–H); AKAP9, VPS13C, and ACSL4 were positively associated with metastatic ability, while HMOX2 was negatively associated with cancer metastasis. Additionally, T stage of SKCM was also significantly associated with VPS13C, ACSL4, and HMOX2 transcription levels (*P* < .05; Fig. 8M–O), and patients with T3 or T4 stages had significantly enhanced HMOX2 expression and decreased VPS13C and ACSL4 expression. These results suggested that our identified hub genes played a vital role in metastasis and progression of SKCM. Therefore, we considered whether these was the reason that our identified genes controlled the overall survival of patients with SKCM and investigated the relationship between clinical features and patient survival. As shown in Figure 8P–S, overall survival was significantly associated with patient age, T stage, TNM stage, and metastatic ability of the cancer (*P* < .01). Intriguingly, patients with primary melanoma or higher tumor stage had significantly worse outcomes, which suggested that AKAP9, VPS13C, ACSL4, and HMOX2 might play a vital role in the overall survival of SKCM through mediating cancer growth and metastasis.

### 3.6 Associations between prognostic risk score and clinical characteristics of SKCM

**Figure 9:**
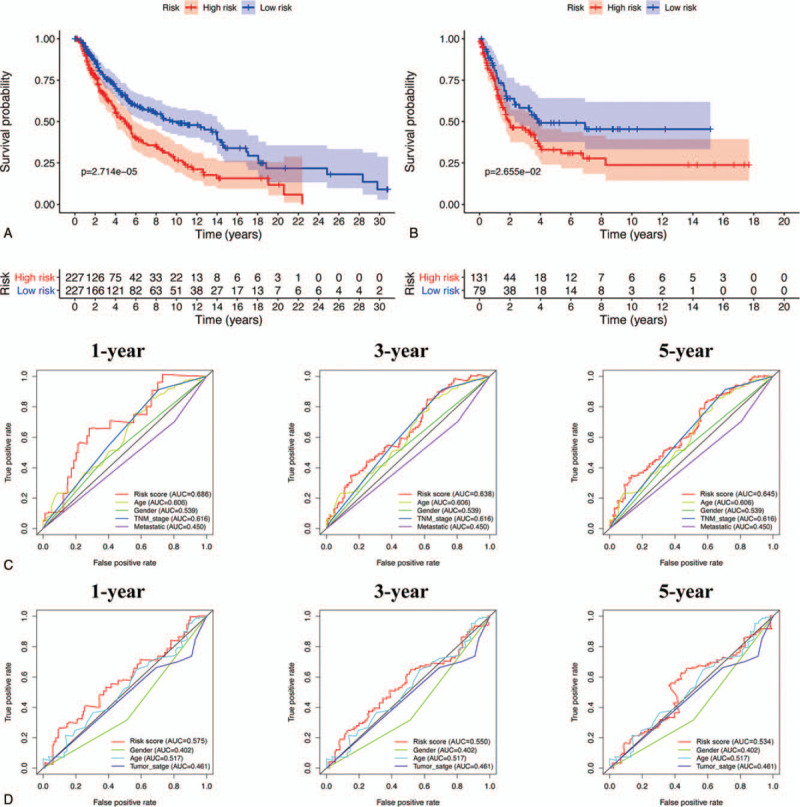
The prognostic value of constructed risk model in SKCM patients. Survival curve of risk model in TCGA (A) and GSE65904 (B) cohort. ROC curves for forecasting overall survival in TCGA (C) and GSE65904 (D) cohort. SKCM = skin cutaneous melanoma, TCGA = the Cancer Genome Atlas.

**Figure 10:**
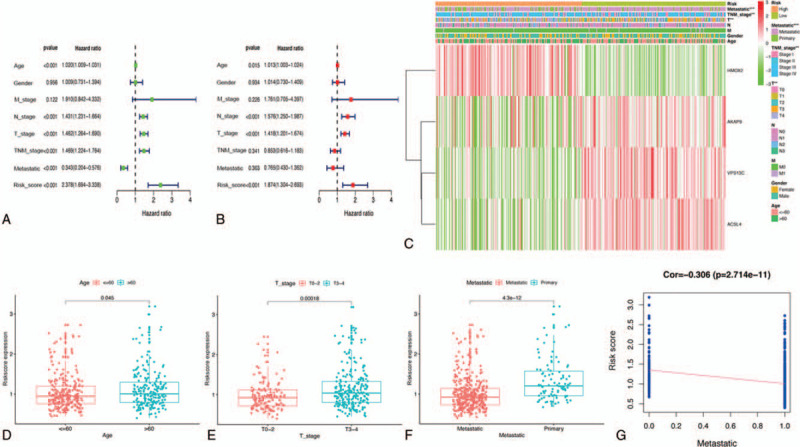
Efficacy evaluation of constructed prognostic model in TCGA cohort. Univariate (A) and multivariate (B) Cox regression analysis of the clinicopathological features. (C) The heatmap shows the distribution of clinicopathological features and OS genes expression in two risk subgroups. (D-F) The relationship between the risk scores and clinicopathological features. (G) Correlation test between the risk score and cancer metastasis. TCGA = the Cancer Genome Atlas.

**Figure 11:**
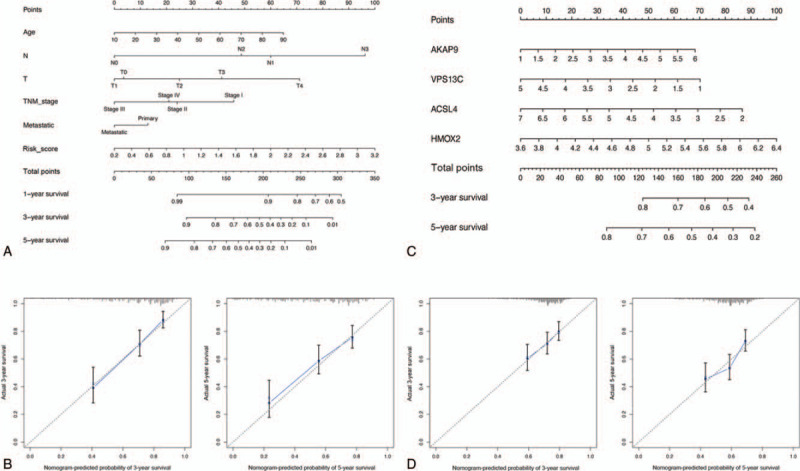
Nomogram construction and conformity examination. (A) The nomogram based on the risk score and other clinical factors was calculated for predicting 1-, 3-, and 5-year survival in SKCM patients. (B) The calibration plot of the risk score-associated nomogram. (C) The nomogram based on 4 hub genes expression level was calculated for predicting 3- and 5-year survival in SKCM patients. (D) The calibration plot of the hub genes-associated nomogram. SKCM = skin cutaneous melanoma.

As per the survival analysis indicated in Figure 9A, overall survival of patients with SKCM was significantly decreased with an increased risk score in TCGA cohort. In addition, ROC analysis indicated that our prediction model was more credible than the clinicopathological characteristics at both 1, 3, and 5 years in TCGA cohort (Fig. 9C). Similar conclusions were also validated in the GSE65904 cohort (Fig. 9B, D), demonstrating the moderate specificity and sensitivity of our prognostic model. Further, univariate and multivariate Cox regression analysis determined that risk score could be identified as an independent prognostic feature associated with SKCM prognosis (Fig. 10A,B). Moreover, we evaluated the connection between risk score and each clinicopathological characteristic, revealing that patients in T0–2 stage or those with metastatic melanoma were significantly associated with lower risk scores (Fig. 10E,F). Meanwhile, risk score was also significantly associated with the age of patients with SKCM (Fig. 10D). Additionally, we discovered that risk score was negatively associated with cancer metastasis (Fig. 10G), indicating that our constructed risk model was significantly associated with SKCM prognosis possibly by predicting the cancer's metastatic ability. A heatmap revealed the expression of the four hub OS genes in the high- and low-risk groups (Fig. 10C), and their expression level in the high-risk group was remarkably consistent with their respective prognostic value in patients with SKCM. Meanwhile, the 2 risk groups differed significantly with respect to T stage, TNM stage, and metastasis in TCGA cohort. A nomogram plot based on risk score and other clinical characteristics was modeled to calculate the clinical outcomes of patients with SKCM (Fig. 11A); calibration plots at 3 and 5 years demonstrated good conformity with our prognostic model (Fig. 11B). A nomogram plot was also developed based on expression of the four hub OS genes, allowing us to calculate the survival probabilities of each patient with SKCM at 1, 3, and 5 years (Fig. 11C). Calibration plots also indicated good conformity between the predicted and observed outcomes at 3 and 5 years (Fig. 11D). These results indicated that our prognostic model showed great promise for predicting SKCM outcomes and clinical features.

### 3.7 GSEA analysis

**Figure 12:**
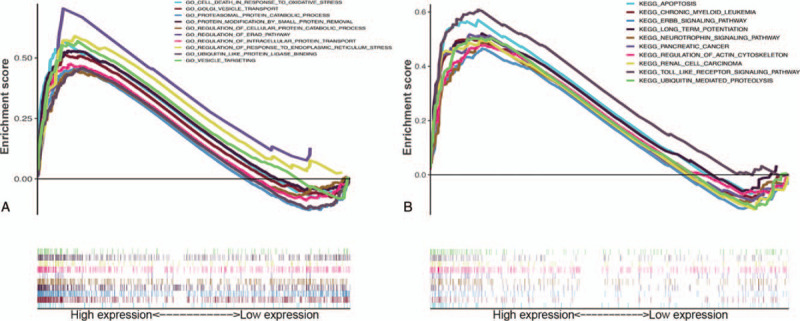
GSEA analysis. (A) Top 10 classes of GO-GSEA enrichment terms. (B) Top 10 classes of KEGG-GSEA enrichment terms. GO = gene ontology, GSEA = gene set enrichment analysis, KEGG = Kyoto Encyclopedia of Genes and Genomes.

GSEA analysis indicated that our selected four hub genes were significantly associated with several GO functions, especially death in response to oxidative stress, Golgi vesicle transport, proteasomal protein catabolic process, protein modification by small protein removal, regulation of cellular protein catabolic process, regulation of the ERAD pathway, regulation of intracellular protein transport, regulation of response to endoplasmic reticulum stress, ubiquitin like protein ligase binding, and vesicle targeting (Fig. 12A). In KEGG pathway analysis, these genes were also significantly associated with apoptosis, chronic myeloid leukemia, the ERBB signaling pathway, long term potentiation, the neurotrophin signaling pathway, pancreatic cancer, regulation of actin cytoskeleton, renal cell carcinoma, the Toll-like receptor signaling pathway, and ubiquitin mediated proteolysis (Fig. 12B).

## 4 Discussion

Melanoma is 1 of the most common skin malignancies, and poor patient prognosis is significantly associated with its high metastatic capacity.^[44]^ Although many novel diagnostic techniques and molecular biomarkers have been discovered in recent years, they have not sufficiently improved early diagnosis and prognosis of SKCM.^[11]^ Therefore, it is imperative to identify SKCM prognosis-related molecules and the exact mechanism of cancer metastasis. In the present study, we constructed a co-expression network of OS genes to identify candidate gene clusters and hub OS genes involved in the prognosis of SKCM. Interestingly, we found that genes in the key module (turquoise) were not only significantly associated with SKCM overall survival but were also associated with the metastatic phenotype. Thus, we speculated that the key module could be employed to predict the outcomes of SKCM. What's more, function analysis indicated that module turquoise was mainly enriched in oxidative stress and oxygen levels, while pathway enrichment analysis suggested this module could possibly play a role in the progression of several tumors, including pancreatic cancer, colorectal cancer, prostate cancer, hepatocellular carcinoma, glioma, endometrial cancer, and especially melanoma.

In the key module, 26 OS genes with MM > 0.6 and GS > 0.2 were screened from 695 module genes. Among them, four differentially expressed prognosis-related OS genes (AKAP9, VPS13C, ACSL4, and HMOX2) were identified as hub genes for further exploration. mRNA and protein expression patterns of these four genes using the expression data from TCGA and HPA cohorts revealed that ACSL4 and HMOX2 were overexpressed, while AKAP9 and VPS13C were downregulated in SKCM tissues. Meanwhile, Kaplan-Meier analysis revealed that HMOX2 was negatively associated with the overall survival of patients with SKCM, while AKAP9, VPS13C, and ACSL4 were positively related to patient outcome. These results might correspond with the modulation effects of these genes in cancer metastasis and growth. Omar et al. determined that AKAP9 acts as a metastasis-promoting gene in SKCM.^[45]^ In addition, as an ROS scavenger in cellular compartments, downregulation of HMOX2 was shown to significantly inhibit tumor growth in lung cancer in vivo.^[46]^ These findings concurred with our results that patients with SKCM had poor prognosis if their cancers were in stage III-IV or in primary sites. Meanwhile, downregulation of HMOX2 or overexpression of AKAP9, VPS13C, and ACSL4 was significantly associated with SKCM metastasis, growth inhibition, and higher overall survival, which was also associated with the prognostic effects of SKCM clinicopathological features. This suggested that our four specific OS genes might provide valuable biomarkers for adjusting treatment strategies of patients with SKCM.

Moreover, to identify whether these specific OS genes could be used as a prognostic factor, we constructed a novel prognostic prediction model based on the four hub genes. To our knowledge, this is the first OS-associated risk model for prognostication. Univariate and multivariate Cox regression analyses revealed that our risk model was an independent prognostic factor and demonstrated reliable prognostic value for SKCM. In addition, survival and ROC analyses confirmed the advantage of its biological implications for predicting SKCM prognosis, and its improved predictive accuracy compared with clinicopathological criteria. Our nomogram analysis confirmed the credibility of the genetic risk signature in predicting the overall survival of patients with SKCM. Taken together, our explorations demonstrate the prognostic value of an OS gene-associated risk model for patients with SKCM and suggest a novel direction for further research.

OS plays a critical role in various stages of carcinogenesis and cancer progression.^[47,48]^ SKCM research has also recently identified that OS may be closely associated with metastasis.^[23]^ Considering the role of our four hub genes in cancer metastasis and growth, we assessed the relationship between risk score and SKCM clinical factors and found that our risk model was closely associated with SKCM metastasis and T stage of cancer. Therefore, our risk model was not only highly correlated with SKCM prognosis but may also provide potential biomarkers for predicting tumor progression.

Nonetheless, there are some limitations in this study. First, this study was designed as a retrospective analysis; more prospective research should be performed to verify our results. Second, our results lack in vitro or in vivo exploration to confirm the reliability of our mechanism analysis. Therefore, we need to conduct several experiments to prove the mechanistic connections between these genes and SKCM progression.

## 5 Conclusion

In conclusion, after constructing a co-expression network and performing bioinformatics analyses, we identified four prognosis-associated OS genes related to metastasis in patients with SKCM. We also successfully constructed a prognostic model with powerful predictive effects. To our knowledge, this is the first OS-associated model to predict the prognosis of malignancies. Our study provides a novel research approach for studying the pathogenesis of SKCM, and greatly contributes to elucidating the metastatic mechanism of SKCM.

## Acknowledgment

We thank the TCGA, University of California Santa Cruz Xena, Genome Tissue Expression, and GSE65904 database for providing all data in this research.

## Author contributions

**Conceptualization:** Jinmin Zhao.

**Data curation:** Xianpei Wu.

**Formal analysis:** Xianpei Wu.

**Methodology:** Xianpei Wu.

**Project administration:** Jinmin Zhao.

**Resources:** Jinmin Zhao.

**Software:** Xianpei Wu.

**Validation:** Jinmin Zhao.

**Visualization:** Xianpei Wu.

**Writing – original draft:** Xianpei Wu.

**Writing – review & editing:** Jinmin Zhao.
